# Significantly Higher Prevalence Rate of Asthma and Bipolar Disorder Co-Morbidity: A Meta-Analysis and Review Under PRISMA Guidelines

**DOI:** 10.1097/MD.0000000000003217

**Published:** 2016-04-01

**Authors:** Ming-Kung Wu, Hung-Yu Wang, Yen-Wen Chen, Pao-Yen Lin, Ching-Kuan Wu, Ping-Tao Tseng

**Affiliations:** From the Department of Psychiatry (MKW, P-YL), Kaohsiung Chang Gung Memorial Hospital and Chang Gung University College of Medicine; Center for Translational Research in Biomedical Sciences (M-KW, P-YL), Kaohsiung Chang Gung Memorial Hospital, Kaohsiung; Department of Psychiatry (H-YW, C-KW, P-TT), Tsyr-Huey Mental Hospital, Kaohsiung Jen-Ai's Home; and Department of Neurology (Y-WC), E-Da Hospital, Kaohsiung, Taiwan.

## Abstract

Asthma and bipolar disorder (BD) are 2 distinct diseases that share similar pathophysiology. This study aimed to determine their relationship thorough a meta-analysis of articles on their comorbidity rate.

The aim of the study is to examine the overall prevalence rate of BD in asthmatic patients and of asthma in BD patients compared to healthy controls.

Electronic research of PubMed and ClinicalTrials.gov was performed.

Articles discussing the prevalence rate of BD in patients with/without asthma and the prevalence rate of asthma in those with/without BD, as well as clinical trials in humans and case-controlled trials or cohort studies, were all included. Case reports or series and nonclinical trials were excluded.

Through a random-effects model, a meta-analysis of the results of 4 studies comparing the prevalence rate of BD in patients with/without asthma, and in 6 studies comparing the prevalence rate of asthma in subjects with/without BD were performed.

There were significantly higher prevalence rates of BD in asthmatic patients than in healthy controls (*P* < 0.001) and of asthma in BD patients than in healthy controls (*P* < 0.001). Only the patient's mean age significantly modulated the odds ratio of the prevalence rate of asthma in BD patients (slope = 0.015, *P* < 0.001).

Only 10 studies were included and most were cross-sectional studies. The possible confounding effect of medication on BD or asthma onset was not investigated. Any possible etiology of the comorbidity was also not determined.

This meta-analysis highlights the importance of the significantly high comorbid rate of BD and asthma, and the positive association with age. Special attention must be given to the comorbidity of asthma and BD, especially in older patients.

## INTRODUCTION

Asthma is a troublesome disease that has a high mortality rate in the world.^1^ Similarly, bipolar disorder (BD) contributes to significant socio-economic burden globally. These 2 belong to distinct disease categories and do not seem to have any association with each other. However, as knowledge on the comorbidity of BD improves, many more comorbid illnesses have been found. In recent reports, asthma has been associated with BD. This is especially important as patients with severe mental illness are believed to have higher comorbidities and higher mortality rates compared to healthy subjects. These patients also have less healthy habits and less medical consults.^2^ Thus, public health policies must be drawn up to address these problems.

Asthma has been initially believed to be a disorder of inflammation.^3^ In asthmatic patients, inflammatory cytokines like interleukin-4 (IL-4), IL-5, and IL-13 are altered along with asthma exacerbation.^4^ Similarly, BD patients are found to have dysfunction of inflammation when the disease is aggravated or subsides.^5,6^ Alterations of IL-4, IL-6, and IL-12 have been proven in BD patients under different emotional states.^7–9^ As such, some researchers suggest that these 2 diseases at least share similar mechanisms in their pathophysiology.^10^

Moreover, in clinical practice, some reports discuss the comorbidity of asthma and BD, either in forms of first onset of asthma or BD. Some have tried to investigate the prevalence rate of asthma in BD patients compared to healthy controls.^11–17^ On the other hand, other studies have compared the prevalence rate of BD in asthmatic patients and in healthy controls.^10,18–20^ Lastly, the medication used in 1 disease may cause a flare up of the other disease. For example, the steroids used in asthma may aggravate the emotional changes in BD.^21^

In clinical studies, there is a significantly higher prevalence rate of asthma in BD patients^11,13–17^ or a significantly higher prevalence rate of BD in asthmatic patients compared to those in health controls.^10,18,20^ However, in another report, there is no significant association between asthma and BD.^12,19^ Such inconsistency may be due to different study designs,^19,20^ different latitudes and hemispheres,^12,14^ or different sex proportion.^10,19^ These findings have implications on clinical practice and public health policy.

The present study aimed to summarize current evidences on the comorbidity rate of asthma and BD through meta-analysis and to investigate any possible association between comorbidity and clinical variables in such patients.

## METHODS AND MATERIALS

### Literature Search and Screening

A previous meta-analysis protocol was followed for the present research strategy.^22^ In the first stage, the identification stage, 2 independent psychiatrists performed a systematic literature search with the electronic database of PubMed and ClinicalTrials.gov. To include as many studies as possible, the most simple keywords of “(asthma) AND (bipolar)” was used in the search process, which was limited to only articles written in English and was conducted on December 29, 2015. The 2 authors then examined the titles and abstracts in the screening stage. Reports that were not related to the prevalence rate of BD and asthma were excluded. Inconsistencies and disagreements in selection were settled through consensus after reading the full text of these studies.

Later, in the stage of eligibility, the remaining studies were re-screened using the inclusion criteria of: (1) articles discussing the prevalence rate of BD in subjects with/without asthma or articles discussing the prevalence rate of asthma in subjects with/without BD; (2) articles on clinical trials in humans; and (3) case-controlled trials or cohort studies. Case reports or series, and nonclinical trials were excluded.

The meta-analysis was divided into 2 parts. The first part was a meta-analysis of articles discussing the prevalence rate of BD in patients with/without asthma. The second was a meta-analysis of articles discussing the prevalence rate of asthma in subjects with/without BD. The primary outcomes were the prevalence rate of BD in patients with/without asthma and the prevalence rate of asthma in patients with/without BD.

**FIGURE 1:**
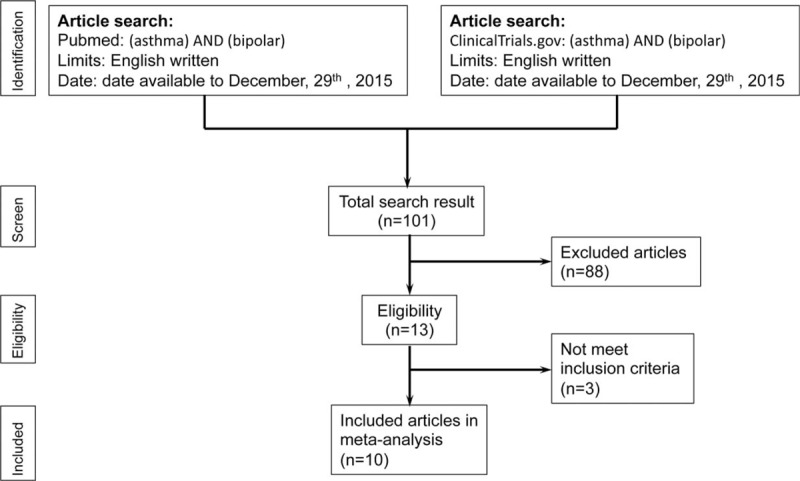
Flowchart of the selection strategy and inclusion/exclusion criteria for current meta-analysis.

All primary outcomes and clinical variables in the studies were extracted as much as possible. If the data were not available, the authors were contacted for the original data. The entire screening and selection process was shown in Figure 1.

### Meta-Analysis and Data Extraction

In the current meta-analysis, the effect size (ES), set as the standardized mean difference based on the odds ratio and treated with a random effects model, was defined as the difference in the prevalence rate of BD in patients with/without asthma and of asthma in subjects with/without BD. An ES > 0 was defined as “favor comorbidity.” If there were no actual case numbers or prevalence rates, or no response from the authors, other statistical parameters such as the *t* or *P* value with the sample size was used to calculate the ES.

The meta-analysis procedures were performed via the platform of the Comprehensive Meta-Analysis software, version 2 (Biostat, Englewood, NJ). Statistical significance was set at a 2-tailed *P* < 0.05. Through Q statistics, related *P* values, and *I*^*2*^ statistics, heterogeneity in the included studies was investigated. At the same time, publication bias was investigated by visual examination of funnel plots and through Egger's regression analysis.^23^ To evaluate the possible confounding effects of clinical variables, subgroup meta-analysis and meta-regression were performed. The meta-regression was performed through the unrestricted maximum likelihood method. The extracted clinical variables for meta-regression included research duration (period of study), mean age, sex proportion (female), age of onset, and race proportion (i.e., African American, Caucasian, Asian, and Native American). The meta-analysis used here fulfilled the criteria of the Preferred Reporting Items for Systematic reviews and Meta-Analyses (PRISMA)^24^ (Supplement Table 1 and Supplement Figure 1). Besides, the ethical approval was not applicable in current study because we would not deal with the patients’ personal data. Furthermore, there were no any patients being harmed due to any procedure in the present study.

## RESULTS

### Studies Included in Each Meta-Analysis

Thirteen articles were initially eligible, but one did not have a control group^25^ and one contained not simply BD patients.^26^ Of the remaining 11 articles, 2 were conducted by the same research team and their clinical data were the same.^11,13^ Thus, only 1 was used to avoid unnecessary duplication. Ten articles were finally included in the meta-analysis (Table 1),^10–12,14–20^ including 4 on the prevalence rate of BD in patients with/without asthma^10,18–20^ and 6 on the prevalence rate of asthma in patients with/without BD.^11,12,14–17^

**TABLE 1:**
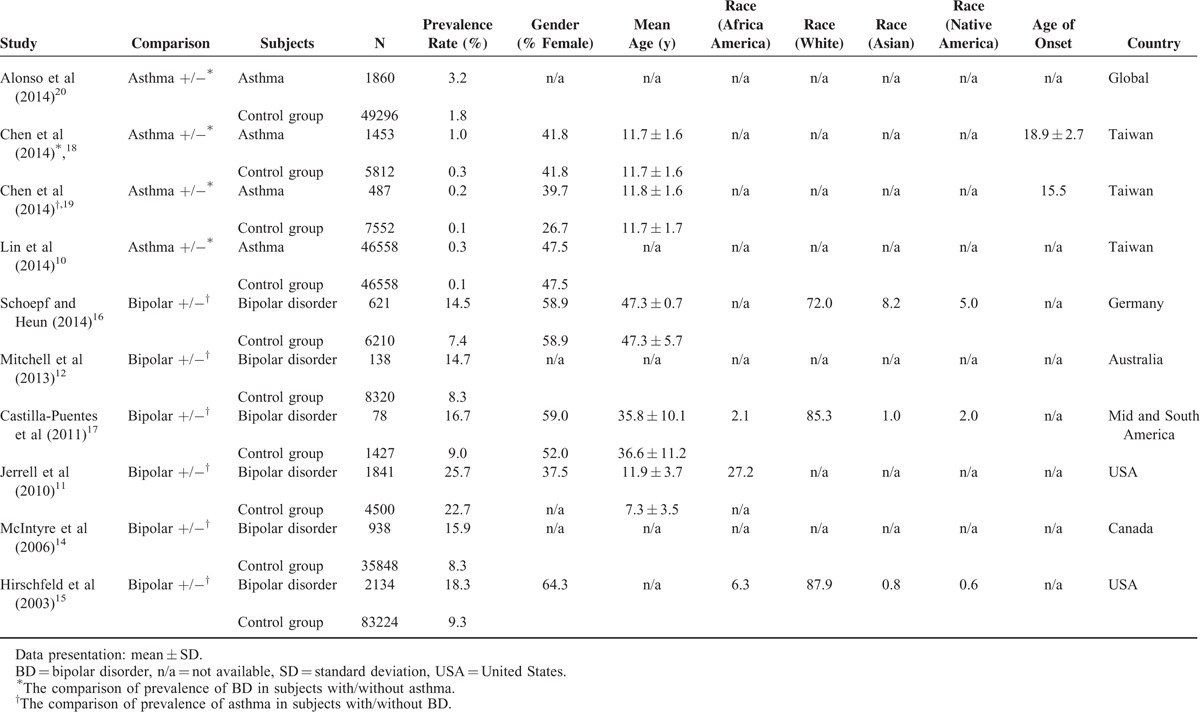
Summary of Characteristics of Studies in Current Meta-Analysis

### Meta-Analysis of the Prevalence Rate of BD in Patients With/Without Asthma

**FIGURE 2:**
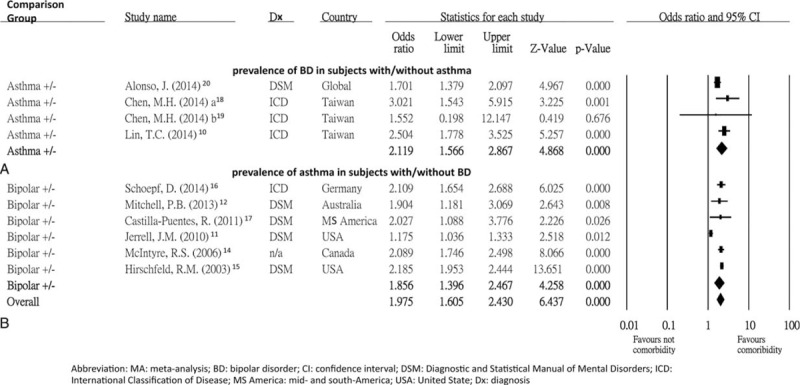
(A) MA of prevalence of BD in subjects with/without asthma; (B) MA of prevalence of asthma in subjects with/without BD. (A) The prevalence of BD was significantly higher in subjects with asthma than those without asthma (*P* < 0.001). (B) The prevalence of asthma was significantly higher in subjects with BD than those without BD (*P* < 0.001). BD = bipolar disorder, CI = confidence interval, DSM = Diagnostic and Statistical Manual of Mental Disorders, Dx = diagnosis, ICD = International Classification of Disease, MA = meta-analysis, MS America = mid- and south-America, USA = United States.

This meta-analysis of 4 studies included 50,358 patients with asthma and 109,218 healthy controls. The prevalence rate of BD was significantly higher in asthmatic patients than in healthy controls (ES: 2.12; 95% confidence interval [CI]: 1.57–2.87; *P* < 0.001) (Figure 2A). There was no statistically significant heterogeneity within the recruited studies (Q = 5.36; *df* = 3; *I*^*2*^ = 44.05%; *P* = 0.147). There was also no significant publication bias detected by Egger's test (*t* = 0.79; *df* = 2; 2-tailed *P* = 0.514) or by visual examination of the funnel plot.

Meta-regression was only performed for the female proportion because of the lack of data. There was no statistically significant association between the prevalence rate of BD in patients with asthma and female sex (*P* = 0.953).

### Meta-Analysis of the Prevalence Rate of Asthma in Patients With/Without BD

Six studies that covered 5750 patients with BD and 139,529 healthy controls were included in this meta-analysis. There was a significantly higher prevalence rate of asthma in patients with BD than that in healthy controls (ES: 1.86; 95% CI: 1.40–2.47; *P* < 0.001) (Figure 2B). However, there was statistically significant heterogeneity within the recruited studies (*Q* = 59.88; *df* = 5; *I*^*2*^ = 91.65%; *P* < 0.001). There was no significant publication bias by Egger's test (*t* = 0.31; *df* = 4; 2-tailed *P* = 0.776) or by visual examination of the funnel plot.

In the meta-regression, there was a significantly positive association between the odds ratio of asthma in patients with BD and mean age (slope: 0.015; *P* < 0.001) rather than female sex proportion or the proportions of Caucasian, Asian, and Native American (*P* = 0.737, 0.797, 0.807, and 0.786, respectively). A meta-regression of African American and duration of research was not performed because of the lack of data.

## DISCUSSION

The current meta-analysis demonstrates that BD and asthma have a significant comorbidity rate with each other. The prevalence rate of asthma is significantly higher in BD patients than in healthy controls. Similarly, the prevalence rate of BD is significantly higher in asthmatic patients than in healthy controls. Among the clinical variables, only mean age has a significantly positive association with the odds ratio of asthma in BD patients.

This meta-analysis provides evidences of the significantly high prevalence rate of comorbid of asthma and BD. In previous reports, evidences reveal that both asthma and BD share common immune abnormalities, such as the abnormal expressions of IL-6^7,8,27,28^ and tumor necrosis factor-α (TNF-α).^5,28^ The high comorbidity between these 2 distinct diseases should be important for clinicians but in clinical practice, there remains a paucity of conclusive evidence as regards the comorbid prevalence rates. This meta-analysis provides the link between these 2 distinct diseases in clinical application, which is especially important because in clinical practice, the physical problems are frequently missed in patients with severe mental illness.^2^ Thus, psychiatrists should be aware of possible comorbid asthma during the treatment of BD. Medication with exacerbating effects on the asthmatic activity, such as beta-blocker, must be avoided.^29^ At the same time, physicians should pay attention to distinguishing the symptoms of asthma and agitation during manic attack and avoid medications, such as steroids, that can induce manic symptoms.^21,30^

Although there is no statistically significant publication bias detected in the present study, most patients are either in countries located at higher latitudes or in the Northern Hemisphere^11,14–16^ or mainly in a few countries such as Taiwan^10,18,19^ and the United States.^11,15^ This may be because the health care system is widely-used and is well-established in these areas.

However, this imbalance in the distribution of countries of the studies may result in some publication bias in the current meta-analysis. The distribution and frequency of asthma vary with climate and air pollution in the environment.^31^ Furthermore, surveillance studies in areas of lower latitudes or in the Southern Hemisphere should be performed.

An interesting finding is the significantly positive association between mean age and the odds ratio of asthma in patients with BD. In previous reports, asthma is believed to be more severe in older than in younger patients^32^ and that there is a higher mortality rate in patients >50 years old.^1^ Moreover, the older the age of asthma onset, the more frequent the steroid treatment is needed.^33^ This phenomenon may be explained by trends in the alteration of specific immune cytokines, such as the reduction in interferon-gamma (IFN-γ) along with aging,^34^ which has been correlated with asthma severity.^35,36^ At the same time, there are changes in specific immune cytokines in patients with BD, such as reduced IFN-γ^6^ and increased IL-4 and TNF-α.^37^

At present, there are no reports that provide a correlation between immunity and the age of patients with BD. Further investigations on the association between age of immunity dysfunction and onset of asthma are therefore warranted in the future.

## LIMITATIONS

This study has some limitations that must be considered. First, the total number of studies included is small, especially in the meta-analysis of the prevalence rate of BD in patients with/without asthma, which may influence the meta-analysis. Second, most of the included studies are cross-sectional studies. In fact, mean age, when available, has a wide range in each study. The mean ages of BD and asthma onset are distinct. This may result in the under-diagnosis of such diseases. Third, the possible confounding effects of medication on the onset of BD or asthma are not investigated. As previously mentioned, some medications such as beta-blockers or steroids can result in symptoms of asthma or mood changes. Furthermore, although there was an attempt to extract most of the clinical variables, the related meta-regression was not performed because of the lack of data. Lastly, only a conclusion of “observation result” was attained in the present study. We could only prove the “comorbidity” rather than “same etiology/pathophysiology.” Therefore, the phenomenon of comorbidity might be derived from similar risk factors or symptomatology, such as anxiety, but not from the same etiology or pathophysiology between these 2 diseases. We lack of the “key” link between these 2 diseases. Therefore, clinicians need to be careful when apply our study in clinical practice.

## CONCLUSION

The current meta-analysis highlights the importance of the significantly high comorbid rate of BD and asthma, and the positive association with age. These findings serve to remind clinicians that special attention should be given to the comorbidity of asthma and BD, especially in older patients.

## Supplementary Material

Supplemental Digital Content
